# Genetic Characterization of Porcine Circovirus Type 2 from Pigs with Porcine Circovirus Associated Diseases in Argentina

**DOI:** 10.5402/2011/560905

**Published:** 2011-05-30

**Authors:** Ariel Pereda, Pablo Piñeyro, Ana Bratanich, María Alejandra Quiroga, Danilo Bucafusco, María Isabel Craig, Javier Cappuccio, Mariana Machuca, Agustina Rimondi, Marina Dibárbora, Hector Ramón Sanguinetti, Carlos Juan Perfumo

**Affiliations:** ^1^Laboratorio Aves y Porcinos, Instituto de Virologia CICVyA, Instituto Nacional de Tecnologia Agropecuaria (INTA), CC25, 1712 Castelar, Buenos Aires, Argentina; ^2^Cátedra de Patología Especial, Departamento de Ciencias Pre-clínicas, Facultad de Ciencias, Veterinarias, Universidad Nacional de La Plata, Casilla de Correo N°296, Calle 60 y 118 S/N, 1900 La Plata, Buenos Aires, Argentina; ^3^Area de Virología, Facultad de Ciencias, Veterinarias, Universidad de Buenos Aires, Chorroarín 280, C1427CWO CABA, Argentina; ^4^Servicio Nacional de Sanidad y Calidad Agroalimentaria, Dirección de Laboratorio Animal, Avenida Fleming 1653, 1640 Martínez, Buenos Aires, Argentina

## Abstract

Porcine circovirus type 2 (PCV-2) has been associated with syndromes grouped by the term porcine circovirus associated diseases (PCVAD). The PCV-2 isolates have been grouped into two major groups or genotypes according to their nucleotide sequence of whole genomes and/or ORF-2: PCV-2b, which have, in turn, been subdivided into three clusters (1A–1C), and PCV-2a, which has been subdivided into five clusters (2A–2E). In the present study, we obtained 16 sequences of PCV-2 from different farms from 2003 to 2008, from animals with confirmatory diagnosis of PCVAD. Since results showed an identity of 99.8% among them, they were grouped within a common cluster 1A-B. This preliminary study suggests a stable circulation of PCV-2b among the Argentinean pig population.

## 1. Introduction

Porcine circovirus (PCV) is a small nonenveloped virus that contains a single-stranded circular DNA of about 1.76 kb. PCV was originally isolated as a noncytopathic contaminant of the PK-15 cell line [1] and was classified as a member of *Circoviridae *family, genus *Circovirus*, based on its morphological and genomic characteristics [2, 3]. 

Two phenotypically different but genetically related strains of PCV have been identified in swine. PCV-1 was first detected as a contaminant of the porcine kidney PK-15 cell line [4] whereas PCV-2 has been associated with postweaning multisystemic wasting syndrome (PMWS) [5, 6], porcine dermatitis and nephropathy syndrome (PDNS) [7], proliferative necrotizing pneumonia [8], and reproductive disorders [9], all of them included by the term porcine circovirus associated diseases (PCVAD) [10]. PMWS is an emerging disease in pigs first described in a swine herd in Canada in 1991 [5] and also endemic in many swine-producing countries. In Argentina, PMWS was first reported in 2002 [11]. 

Diagnosis of PMWS is based on the presence of compatible clinical signs [12], characteristic histopathological lesions, and detection of PCV-2 antigen within typical lesions [10, 13].

The genomic structure of PCV-2 consists of two intergenic regions flanked by three open reading frames (ORFs): ORF-1, which encodes two replication proteins (Rep and Rep′), ORF2, which encodes the Cap protein containing the immunoreactive epitopes and is more variable at nucleotide sequence than ORF1, and ORF3, which encodes a proapoptotic protein [14]. Currently, PCV-2 genotype definition and nomenclature has been proposed according to the genome sequence of the whole genome and/or ORF2 [15, 16]. Five genotypes have been identified to date [17, 18]: PCV-2a and PCV-2b, which correspond to the main phylogenetic groups, PCV-2c, which has been described only in Denmark, and PCV-2d and 2e, which have been described in China [18]. PCV-2a has been further subdivided into five clusters (2A–2E) [15] and PCV-2b into three clusters (1A–1C). Since 2005, high mortality outbreaks of PMWS reported in North America have been associated with PCV-2b [19, 20]. In South America, only Brazil has carried out genotyping studies on PCV-2. Phylogenetic studies have shown that PCV-2 isolates belong to PCV-2b and PCV-2a [21, 22]. The objectives of this study were to compare the nucleotide and amino acid sequences of the PCV-2 ORF2 identified in lymph nodes from pigs with PCVAD from different herds of Argentina between 2003 and 2008.

## 2. Materials and Methods

### 2.1. Immunolabelling for PCV-2

Immunohistochemistry was performed with a polyclonal anti-PCV-2 antibody (VMRD, Inc., WA, USA, 210-70 PCRV). Briefly, tissue sections were deparaffinized with xylene and rehydrated through graded alcohols. Slides were flooded for 15 min with 3% H_2_O_2_ to remove endogenous peroxidase activity. Tissues were rinsed for 5 min in 0.1 M PBS (pH 7.5) and then incubated with preheated 0.05% protease XIV for 40 minutes. Tissue sections were rinsed in PBS and flooded with 0.5% skim milk in PBS for 20 min at room temperature. PCV-2 antibody was used at a 1/200 dilution in 0.1 M PBS, and incubated for 1 hour at 37°C. Biotinylated G protein (1/500) was used as a secondary antibody and was incubated for 40 min at room temperature. Streptavidin-peroxidase (LSAB2 System HRP K0673, DAKO Laboratories Co., CA, USA) was applied for 15 min at room temperature. Sections were finally incubated in diaminobenzidine-hydrogen peroxide solution for 8 min and counterstained with Harris' haematoxylin. Positive and negative controls were used. Immunohistochemistry were graded based on the intensity of immunolabelling as follows: + = slight, ++ = moderate, and +++ = abundant.

### 2.2. Samples and DNA Extraction

The samples used in this study corresponded to pig submissions to the Diagnostic Pathology Service, Faculty of Veterinary Science, La Plata National University, Argentina, from 2003 to 2005, and to the Virology laboratory from the Faculty of Veterinary Science of Buenos Aires from 2007 to 2008. These samples originated from 101 lymph nodes from pigs (36 to 140 days old) with confirmatory PCVAD diagnosis by immunohistochemistry (IHC). Samples from 2003 to 2005 were pooled in nine groups (6–10 samples/pool) according to the farm, province, and year of collection. Samples from 2007 to 2008 were processed individually (Table 1). Samples were homogenized with a mortar and pestle, in some cases after deparaffinization with xylene, and kept at −80°C until processed.

DNA extraction was carried out using the QIAamp DNA mini kit (Qiagen, Valencia, USA) in accordance with the manufacturer's instructions.

### 2.3. Identification of PCV-2 and Amplification of the ORF2

PCV-2 specific DNA was amplified by PCR with GoTaq (Promega) in a MyCycler thermocycler (BioRad, UK). The ORF2 from positive samples was further amplified from nucleotides 998 to 1757 (PCV-2b genome; GenBank Accession Number, AF112862), using the protocol reported by Fort et al. [23]. In this protocol, two internal primers (capARv 5′-ACCCTTTGAATACTACAGA-3′ and capBFw 5′-GGGAGGAGTAGTTTACATA-3′) were added to make the sequencing process more reliable, since it amplifies the whole ORF2 in two partially overlapping fragments. Cycling conditions were as follows: 94°C 5 min, 35 cycles of 45 sec at 94°C, 45 sec at 42°C and 1 min at 72°C, and a final extension cycle of 7 min at 72°C.

### 2.4. Nucleotide Sequencing and Phylogenetic Analyses

ORF2 amplified fragments were sequenced with the BigDye terminator kit (Applied Biosystems, Foster City, CA, USA) on an ABI 3500xL Genetic Analyzer (Applied Biosystems, Foster City, CA, USA). Sequence information was derived from overlapping sequences covered by forward and reverse primers. Sequences are available at GenBank, accession numbers EU980087 through EU980094. Then, all available isolates obtained were included in a multiple alignment using CLUSTAL X Version 1.8.3 program [24], and the percent identities were calculated. The phylogenetic trees, calculated by the neighbor-joining method, were computed with the DNADIST and NEIGHBOR modules of the PHYLIP package [25]. Bootstrapping values (1000 replicates) were calculated with the SEQBOOT, DNADIST, NEIGHBOR, and CONSENSE modules. Branches with bootstrapping values ≥70 were considered significant, corresponding to a confidence interval ≥95% [26]. For visualization and printing of the trees, the TREEVIEW program, Version 1.6.6 was used [27]. To root the phylogenetic tree, we used the sequence of a porcine circovirus 1 (GenBank accession number AY184287).

## 3. Results

### 3.1. Detection of PCV-2 by PCR

The signs that predominated in the affected pigs were principally wasting in which the animals presented slow growth, lethargy, anorexia, cachexia, diarrhea, and dyspnea (Table 1). From those animals, 101 lymph nodes were processed and 58 (57.4%) were positive for PCV-2 by IHC. Samples from 2003 to 2005 were grouped in 9 pools and 8 (88.8%) were positive for PCV-2 by PCR. This correlates with the lack of IHC positive samples in the negative pool. Seven samples from 2007 and 2008 were processed individually and all resulted positive by PCR.

### 3.2. Nucleotide and Amino Acid Sequence of ORF2

The entire Cap coding region of 16 Argentinean samples was amplified and sequenced (Table 1). The sequence analysis showed that they contained 699 nt, and no gaps were detected. Comparison of the sequences analyzed revealed an identity of 99.8% among them. All sequences presented the typical PCV-2 1486 motif TcAaacCCC/CGC. At the amino acid level, only two polymorphic sites were detected in the following samples: in 7109 we found K63R and in 7013, 7108, and 7198 we found T190A. Only the K63R mutation was localized in an immunogenic domain, specifically at the domain A. 

These sequences were aligned with cap gene sequences from PCV-2 strains available in the GenBank database from different countries. All Argentinean amino acid sequences showed high identity with the sequences grouped in genotype 2b.

### 3.3. Phylogenetic Analysis

**Figure 1:**
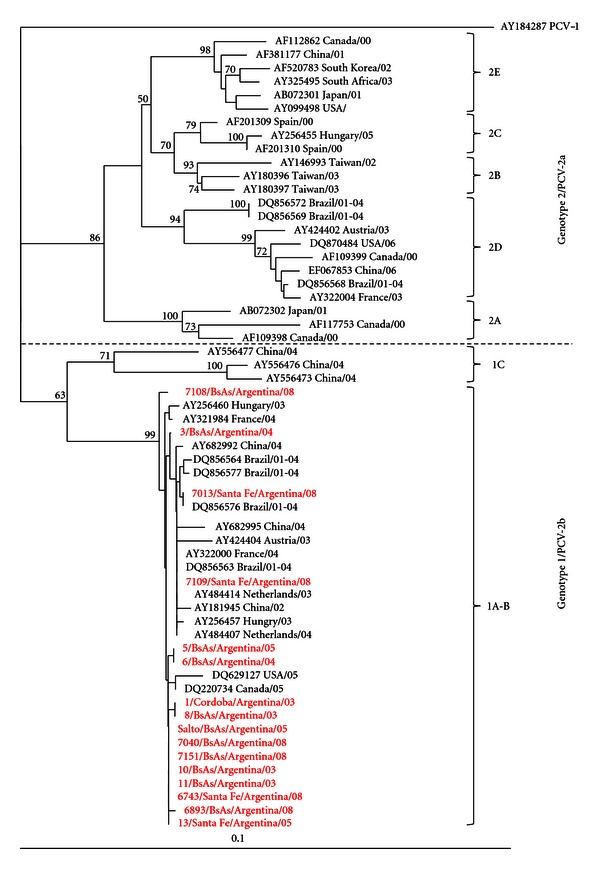
Phylogenetic tree calculated on the basis of ORF2 sequences of porcine circovirus 2 (PCV-2) using the Neighbor Joining algorithm of the PHYLIP package [25] with 1000 bootstrap replicates. Bootstrap values over 500 are shown as 1/10 of the value. Argentinean isolates are identified in red. Reference strains are indicated with the corresponding GenBank accession number and the country and year of report. Isolates are grouped by subgenotypes as described by Fort et al. 2007 [23] and separated by subgroups by the dashed line as described by Wiederkehr et al. 2008 [28].

The phylogenetic tree obtained with the Argentinean and reference PCV-2 strain sequences showed two main clusters wellsupported by bootstrap analysis (Figure 1). All the Argentinean sequences analyzed grouped together in one cluster and were closely related to sequences from genotype PCV-2b isolates, and more specifically to cluster 1A-B. In this last case, the low bootstrap values made the differentiation of the two clusters uncertain. We propose that they should be joined in a common cluster 1A-B. This cluster grouped also isolates from Europe (Hungary, France, Austria, and the Netherlands), Asia (China), and North America (USA and Canada). In the other cluster, named 1C, only grouped sequences from Asia (China).

## 4. Discussion

Recent phylogenetic analysis of complete PCV-2 genomic sequences obtained from GenBank [15] and full-length ORF2 sequences [16] resulted in two major genogroups: 2a and 2b, which are circulating worldwide, 2c, which have been sporadically reported in the 80s [17], and 2d and 2e, recently described in China [18]. The significance of these differences in terms of disease and/or geographic location is currently unknown. However, the severe re-emergence of PCVAD in North America in 2005 coincided with the identification of the PCV-2b genotype [19]. In addition, an epizootic of PMWS in Switzerland was found to be associated with PCV-2b while PCV-2a was identified in single cases of PMWS prior to 2003 [28]. Furthermore, experimental studies in gnotobiotic pigs with or without immunostimulation using PCV-2b alone have shown only moderate lesions in various organs, thus indicating that PCV-2b-stimulated pigs became infected but did not progress to a disease state [29]. However, another study using gnotobiotic pigs and cell culture-derived PCV-2a and PCV-2b clones showed that both were able to induce severe disease in this model [30]. A study has highlighted the importance of the order of infection in which gnotobiotic pigs inoculated with PCV-2b seven days after a PCV-2a infection, but not vice versa, cause PMWS [29]. However, when SPF instead of gnotobiotic pigs was used, results were negative [31]. Unfortunately, sequence studies with PCV-2a followed with PCV-2b in conventional pigs are still lacking.

The above results are indicative of sometimes contradicting information regarding PCV-2 genotyping and its relationship with disease condition. Although it is generally believed that the causes of illness depend on unknown factors, more information related to PCV-2 genotyping could help for a better understanding of the pathogenic mechanisms of PCV-2. 

In the present study, PCV-2 sequences obtained from 16 different farms belonging to the main pig-producing provinces of Argentina were analyzed to determine the PCV-2 genotype. Only samples from 2003 to 2008 with confirmatory diagnosis of PCVAD were processed. Sequence and phylogenetic analyses clustered the PCV-2b group with a common A-B subgroup, with an identity of 99.8% [15]. The reason we named a new cluster A-B is because bootstrap values were not suitable enough, based on the sequences used in this study, to support the differentiation between clusters A and B. The PCV-2b sequences identified were very similar to an isolate previously reported in Argentina in 2005 (Genbank EF458306). The Argentinean PCV-2b genotype isolates were found to be closely related to some isolates from Brazil 2004, North America 2005, Europe 2003-2004, and China 2002. In addition, all sequences present the 1486 motif TcA/aac/CCC/CGC belonging to the PCV-2b genotype described by Cheung et al. 2007 [20], and contain amino acid substitutions similar to those described by Grau-Roma et al. 2008 [16]. In Canada, PCV-2b isolates from PMWS outbreaks in 2005 share an interval identity between 99% and 100% [19]. However, other studies have identified distinctive genotypes from PCVAD samples [28, 32] and a variable identity within each genotype [22].

In Argentina, the first outbreak of PMWS was reported in 2002 [11] and thereafter clinical PCVAD emerged as emerging diseases. Our study comprised PCVAD samples taken from 2003 to 2008 and the results suggest that PCV-2b is the predominant genotype in Argentina and that it could be associated with a systemic form of PCVAD, namely, PMWS. In Argentina, serological or immunohistochemical retrospective studies on PCV-2 have not been carried out and, therefore it remains unknown how and when PCV-2b was introduced in the Argentinean herds. Other sources of infection such as inappropriate used of vaccines can be ruledout because only inactivated PCV-2 and PCV-2 ORF2 protein vaccines have been licensed. 

In summary, the present study contributes to the knowledge on the distribution of PCV-2 genotypes circulating in Argentina. Our findings may also help to establish a base of information to study the emergence of new viral variants in this region.

## Figures and Tables

**Table 1:** PCV-2-positive samples and sample pools included in this study.

Identification	Province location	Main clinical signs associated with PCVAD	Year	Accession number
1*	Cordoba	Wasting	2003	EU980089
3*	Buenos Aires	Cachexia	2004	EU980094
5*	Buenos Aires	Cachexia, dyspnoea	2005	EU980087
6*	Buenos Aires	Diarrhea, dyspnea, pallor	2004	EU980088
8*	Buenos Aires	Dyspnoea	2003	EU980090
10*	Buenos Aires	Wasting	2003	EU980092
11*	Buenos Aires	Wasting, diarrhea, nervous signs	2003	EU980093
13*	Santa Fé	Dyspnoea, fever	2005	EU980091
Salto	Buenos Aires	Wasting	2005	EF458306
6743	Santa Fé	Wasting	2007	HM565918
6893	Buenos Aires	Wasting	2007	HM565920
7013	Santa Fé	Wasting	2008	HM565921
7040	Buenos Aires	Wasting	2007	HM565922
7108	Buenos Aires	Wasting	2007	HM565923
7109	Santa Fé	Wasting	2008	HM565924
7151	Buenos Aires	Wasting	2008	HM565925

*Corresponds to sample pools.

## References

[1] Tischer I, Gelderblom H, Vettermann W, Koch MA (1982). A very small porcine virus with circular single-stranded DNA. *Nature*.

[2] Lukert P, De Boer GF, Dale JL (1995). The circoviridae. *Virus Taxonomy Sixth Report of the International Committee on Taxonomy of Viruses*.

[3] Meehan BM, Creelan JL, McNulty MS, Todd D (1997). Sequence of porcine circovirus DNA: affinities with plant circovirses. *Journal of General Virology*.

[4] Tischer I, Rasch R, Tochtermann G (1974). Characterization of papovavirus and picornavirus like particles in permanent pig kidney cell lines. *Zentralblatt für Bakteriologie*.

[5] Harding JC Post-weaning multisystemic wasting syndrome (PMWS): preliminary epidemiology and clinical presentation.

[6] Ellis J, Krakowka S, Lairmore M (1999). Reproduction of lesions of postweaning multisystemic wasting syndrome in gnotobiotic piglets. *Journal of Veterinary Diagnostic Investigation*.

[7] Segales J, Domingo M, Latimer KS Porcine circovirus is present in cases of porcine dermatitis and nephropathy syndrome (PDNS).

[8] Segales J, Rosell C, Domingo M (2004). Pathological findings associated with naturally acquired porcine circovirus type 2 associated disease. *Veterinary Microbiology*.

[9] Cariolet R, Blanchard B, Le Dimna M Consequences of PCV-2 experimental infection of non immune SPF sows using the intrauterine route.

[10] Opriessnig T, Meng XJ, Halbur PG (2007). Porcine circovirus type 2-associated disease: update on current terminology, clinical manifestations, pathogenesis, diagnosis, and intervention strategies. *Journal of Veterinary Diagnostic Investigation*.

[11] Sarradell J, Perez AM, Andrada M, Rodriguez F, Fernandez A, Segales J (2002). PMWS in Argentina. *Veterinary Record*.

[12] Harding JC, Clark EG (1997). Recognizing and diagnosing postweaning multisystemic wasting syndrome (PMWS). *Journal of Swine Health and Production*.

[13] Rosell C, Segales J, Plana-Duran J (1999). Pathological, immunohistochemical, and in-situ hybridization studies of natural cases of postweaning multisystemic wasting syndrome (PMWS) in pigs. *Journal of Comparative Pathology*.

[14] Mankertz A, Caliskan R, Hattermann K (2004). Molecular biology of Porcine circovirus: analyses of gene expression and viral replication. *Veterinary Microbiology*.

[15] Olvera A, Cortey M, Segales J (2007). Molecular evolution of porcine circovirus type 2 genomes: phylogeny and clonality. *Virology*.

[16] Grau-Roma L, Crisci E, Sibila M (2008). A proposal on porcine circovirus type 2 (PCV2) genotype definition and their relation with postweaning multisystemic wasting syndrome (PMWS) occurrence. *Veterinary Microbiology*.

[17] Segalés J, Olvera A, Grau-Roma L (2008). PCV-2 genotype definition and nomenclature. *Veterinary Record*.

[18] Cui W, Xin G, Ge X, Yang H Genotype analysis of Chinese porcine circovirus type 2 in 2008-2009.

[19] Gagnon CA, Tremblay D, Tijssen P, Venne MH, Houde A, Elahi SM (2007). The emergence of porcine circovirus 2b genotype (PCV-2b) in swine in Canada. *Canadian Veterinary Journal*.

[20] Cheung AK, Lager KM, Kohutyuk OI (2007). Detection of two porcine circovirus type 2 genotypic groups in United States swine herds. *Archives of Virology*.

[21] De Castro M, Cortez A, Heinemann MB, Brandao PE, Richtzenhain LJ (2007). Genetic diversity of Brazilian strains of porcine circovirus type 2 (PCV-2) revealed by analysis of the cap gene (ORF-2). *Archives of Virology*.

[22] Ciacci-Zanella JR, Simon NL, Pinto LS (2009). Detection of porcine Circovirus type 2 (PCV2) variants PCV2-1 and PCV2-2 in Brazilian pig population. *Research in Veterinary Science*.

[23] Fort M, Olvera A, Sibila M, Segales J, Mateu E (2007). Detection of neutralizing antibodies in postweaning multisystemic wasting syndrome (PMWS)-affected and non-PMWS-affected pigs. *Veterinary Microbiology*.

[24] Thompson JD, Higgins DG, Gibson TJ (1994). CLUSTAL W: improving the sensitivity of progressive nultiple sequence alignment through sequence weighting, position-specific gap penalties and weight matrix choice. *Nucleic Acids Research*.

[25] Felsenstein J (1989). PHYLIP* phylogeny interference package (version 3.2). *Cladistics*.

[26] Hillis DM, Bull JJ (1993). An empirical test of bootstrapping as a method for assessing confidence in phylogenetic analysis. *Systematic Biology*.

[27] page RD (1996). Treeview: an application to display phylogenetic trees on personal computers. *Computer Applications in the Biosciences*.

[28] Wiederkehr DD, Sydler T, Brugnera E, Pospischil A, Buergi E, Sidler X Pathogenic differences of porcine circovirus type 2 genotypes in Switzerland.

[29] Harding JC, Ellis JA, McIntosh KA, Krakowka S (2010). Dual heterologous porcine circovirus genogroup 2a/2b infection induces severe disease in germ-free pigs. *Veterinary Microbiology*.

[30] Lager KM, Gauger PC, Vincent AL, Opriessnig T, Kehrli ME, Cheung AK (2007). Mortality in pigs given porcine circovirus type 2 subgroup 1 and 2 viruses derived from DNA clones. *Veterinary Record*.

[31] Opriessnig T, Prickett JR, Madson DM (2010). Porcine circovirus type 2 (PCV2)-infection and re-inoculation with homologous or heterologous strains: virological, serological, pathological and clinical effects in growing pigs. *Veterinary Research*.

[32] Chae JS, Choi KS (2010). Genetic diversity of porcine circovirus type 2 from pigs in Republic of Korea. *Research in Veterinary Science*.

